# Enzymatic Conversion of Xylan Residues from Dilute Acid-Pretreated Corn Stover

**DOI:** 10.1007/s12010-012-9786-5

**Published:** 2012-07-31

**Authors:** Joseph Shekiro, Erik M. Kuhn, Michael J. Selig, Nicholas J. Nagle, Stephen R. Decker, Richard T. Elander

**Affiliations:** 1 National Bioenergy Center, National Renewable Energy Laboratory, Golden, CO 80401 USA; 2 Biosciences Center, National Renewable Energy Laboratory, Golden, CO 80401 USA

**Keywords:** Cellulosic ethanol, Xylan, Xylose, Xylanase, Lignocellulosic biomass, Enzyme conversion, Xylo-oligomer, Xylo-oligomer conversion

## Abstract

Enzymatic conversion of oligomeric xylose and insoluble xylan remaining after effective pretreatment offers significant potential to improve xylan-to-xylose yields while minimizing yields of degredation products and fermentation inhibitors. In this work, a commercial enzyme cocktail is demonstrated to convert up to 70 % of xylo-oligomers found in dilute acid-pretreated hydrolyzate liquor at varying levels of dilution when supplemented with accessory enzymes targeting common side chains. Commercial enzyme cocktails are also shown to convert roughly 80 % of insoluble xylan remaining after effective high-solids, dilute acid pretreatment.

## Introduction

The goal of reducing cellulosic ethanol process cost drives the continued pursuit of higher conversion of xylan to xylose. As dilute acid pretreatment technology has matured, increased focus has been placed on xylan preservation and minimization of yield loss to degradation products. Consequently, pretreatment severity has decreased, leaving significant proportions of original xylan in the form of insoluble xylan and soluble xylo-oligomers. As such, a need has arisen for supplementary means of converting xylan and xylo-oligomers to monomeric xylose. Enzyme catalyzed hydrolysis of xylo-oligosaccharides and insoluble xylan remaining after dilute-acid pretreatment offers significant potential to increase total xylose yield without further degradation product formation.

Previous studies exploring bench-scale, dilute acid pretreatment of corn stover have demonstrated xylose yields approaching 85–90 % of theoretical [23, 25]. However, pilot-scale, dilute acid pretreatment of corn stover found monomeric xylose yields of only 75 %, with upwards of 10 % of the original xylan lost to degradation products, some of which are inhibitory in downstream fermentation [14]. The depression in pretreatment yield at pilot scale, particularly in continuous systems, is likely due to several issues, including residence time control. Lower severity pretreatments focused on the minimization of product degradation have resulted in increased proportions of xylan remaining as xylo-oligomers and insoluble xylan after pretreatment, inspiring the pursuit of a variety of supplemental methods of xylan conversion. Such techniques include a mild secondary thermochemical pretreatment, enzymatic conversion of xylo-oligomers, enzymatic conversion of insoluble xylan, and heterogeneous catalysis [1, 4, 9, 13, 20].

Enzymatic conversion of post-pretreatment xylan and xylo-oligomers is promising because of the potential to produce significant amounts of monomeric xylose while reducing degradation product and inhibitor formation [1]. However, numerous studies have demonstrated the detrimental effects of acetylation and ferulyation on the enzymatic hydrolysis of xylan [5–8, 10, 11, 16]. Similarly, xylo-oligomers remaining after pretreatment and enzymatic saccharification are highly substituted with ferulic acid, diferulates, acetic acid, galactose, arabinose, and uronic acids [2]. Appeldoorn further postulated that these oligomers were recalcitrant due to the presence of these side-chains. Helm et al. [6] hypothesized that the oligosaccharides remaining after effective pretreatment and secondary thermochemical hydrolysis contain a high proportion of 4-*O*-methylglucuronic acids. Supplementing endo-xylanase and beta-xylosidase with accessory debranching enzymes has been shown to improve the enzymatic conversion of native arabinoxylan to monomeric xylose [3, 12, 13, 15, 17, 20].

The current work demonstrates the effectiveness of enzymes in converting xylo-oligomers and insoluble xylan to xylose using pilot-scale dilute acid-pretreated corn stover. A commercial enzyme cocktail was used to demonstrate the conversion of insoluble xylan remaining after pretreatment to monomeric xylose. The effects of product inhibition on xylanses and other enzymes are demonstrated using the hydrolyzate liquor at xylose concentrations of up to 89 g/L. Xylan-to-xylose yields from enzymatic hydrolysis were also enhanced by supplementing a commercial enzyme cocktail with specific debranching enzymes. Addition of these enzymes only increased the overall enzyme usage by 6 % over cellulase loaded at 20 mg protein per gram cellulose.

## Materials and Methods

### Feedstock

Pioneer maize variety 33A14 whole stover from Wray, Colorado was tub ground at the Kramer farm in Wray, Colorado and stored in a semi-trailer in Arvada, Colorado without temperature control. It was further milled at the National Renewable Energy Laboratory (NREL) through a Mitts & Merrill Model 10 × 12 rotary knife mill (Saginaw, MI) to pass a 6.4-mm (0.25-in.) round screen.

### Pre-impregnation

Milled corn stover used in pilot-scale pretreatment experiments was pre-impregnated in a recirculating atmospheric pressure impregnation system previously described [22]. Seventeen kilograms of dry biomass was loaded into a Hastelloy C-276 20-mesh wire basket and immersed in 120 kg of 2.00 % (*w*/*w*) sulfuric acid at 50 °C (±5 °C) for 120 min. The basket was then raised above the tank by a hoist to drain free liquid. Feedstock was further dewatered to 45 % solid content by a 25.4-cm (10-in.) hydraulic press operated at a pressure of 440 psig (3.03 MPa). Samples of the acid-impregnated corn stover were taken for compositional analysis. Finished material was stored at 4 °C until time of use, typically less than 3 days. Composition of the acid impregnated biomass differs from that of native corn stover due to the loss of the extractives fraction during the impregnation process, which includes soluble sucrose and ash. Results of compositional analysis are shown in Table 1 below.

**Table 1:** Compositional analysis of native milled corn stover and 2 % acid impregnated biomass

Component	Initial corn stover (%)	Acid-impregnated biomass (%)
Glucan	34.0 ± 0.8	39.3 ± 0.8
Xylan	22.0 ± 0.6	24.7 ± 0.6
Galactan	1.6 ± 0.1	1.5 ± 0.3
Arabinan	3.1 ± 0.1	3.0 ± 0.4
Sucrose	4.0 ± 0.4	0
Lignin	12.4 ± 0.1	18.0 ± 0.3
Acetyl	2.9 ± 0.1	2.8 ± 0.3
Ash	7.1 ± 1.0	3.9 ± 0.9
Protein	1.6 ± 0.2	ND
Extractives	8.3 ± 0.4	0
Total	96.87 ± 0.67	93.13 ± 0.84

Standard deviations are based on triplicate analysis

*ND* none detected

### Continuous Scale Pretreatment

**Fig. 1:**
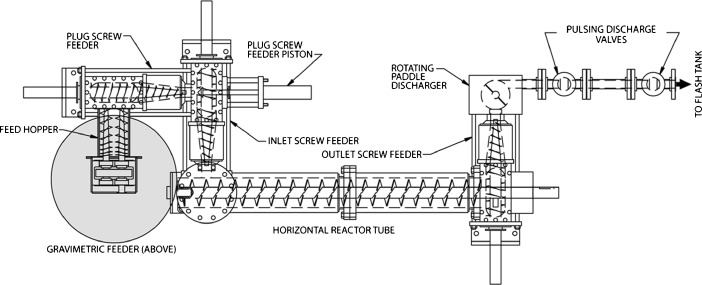
Overhead view of the 200 kg/day horizontal continuous pretreatment reactor

Pretreated materials for this study were produced in the 200-kg/day continuous, high-solids, pilot-scale horizontal pretreatment reactor system (Metso Paper, Norcross, GA) as shown in Fig. 1.

**Fig. 2:**
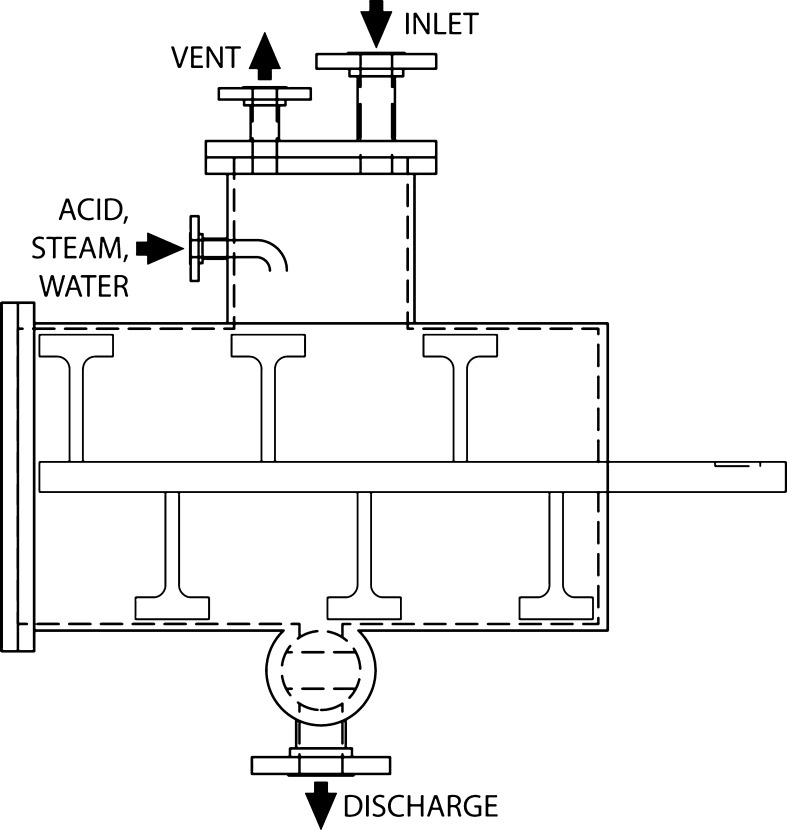
Elevation view of the multipurpose flash tank and batch reactor vessel. Paddles direct material towards reactor center, where discharge port is located. Reactor paddles are equipped with Teflon-lined sweepers to minimize accumulation of material on reactor walls

The design of the 130 L Jaygo (Union, NJ) flash receiver and secondary-oligomer hydrolysis reactor is pictured in Fig. 2. The pretreated slurry was generated over a period of 4 days using pre-impregnated corn stover, at 2.0 % (weight H_2_SO_4_/total liquid weight) acid, 30 % solids loading, a reaction temperature of 158 °C, and a residence time of approximately 5 min. During steady-state operation, the pretreated hydrolyzate was collected in the Jaygo reactor and held below 60 °C.

### Thermochemical Oligomer Conversion

Secondary hydrolysis experiments were conducted in the flash vessel by collecting material produced by the horizontal pretreatment reactor for 120–300 min, taking a whole slurry sample, and then isolating the vessel.

The process slurry was immediately heated to 130 °C indirectly by the steam jacket. Additional acid (10 % (*w*/*w*) sulfuric acid solution) was added to achieve a loading of 15.6 g acid/kg dry biomass (in addition to the sulfuric acid loading used in horizontal pretreatment reactor operation), and the reactor was held at 130 °C for 20 min while agitating at 30 rpm. At the end of the oligomer conversion reaction time, the vessel was brought to atmospheric pressure over the span of 1 min by controlled venting to a 15 °C condenser, where flash condensate was collected separately. Cooling water was also applied to the vessel jacket following the flash steps. Samples of the pretreated slurry were collected and analyzed as described earlier. Compositional analysis of this material after the thermochemical oligomer conversion stage is shown in Table 2 below.

**Table 2:** Compositional data from pooled 200 kg/day horizontal pretreatment reactor runs

Solids Composition	Ash	Protein	Lignin	Glucan	Xylan	Galactan	Arabinan	Fructan	Acetate
Component (% *w*/*w*)	5.9	0.0	27.7	61.4	4.1	0.7	1.1	0.0	1.1
Raw liquor data	Cellobiose	Glucose	Xylose	Fructose	Arabinose	Galactose	Acetic acid	HMF	Furfural

Monomeric sugars (g/L)	1.1	15.2	89.1	1.0	11.4	5.9	15.1	0.5	3.4
Oligomeric sugars (g/L)	–	2.3	9.3	1.7	0.3	0.5	–	–	–
Total sugars (g/L)	–	17.5	98.4	2.7	11.7	6.4	–	–	–

Analysis of total sugars derived from 4 % acid hydrolysis with sugar recovery standards and subsequent monomeric sugar analysis

*HMF* hydroxymethylfurfural

### Enzymatic Hydrolysis of Washed Pretreated Corn Stover Solids

A representative pretreated corn stover sample from the 200-kg/day horizontal pretreatment reactor run and mild thermochemical oligomer conversion step in the Jaygo reactor was hydraulically pressed to remove the hydrolyzate liquor. The pressed solids were then washed to remove soluble compounds prior to enzymatic hydrolysis. To represent a process-relevant solid/liquid separation, 5 % of the unconditioned liquor was added back to the washed solids to simulate the hydrolyzate liquor that would remain in the solids following a separation using a reasonable amount of wash water.

The enzymatic hydrolysis of the washed solids was performed with the Novozymes (Bagsvaerd, Denmark) Cellic CTec 2 enzyme preparation at a temperature of 48 °C and a pH of 5.0, at an enzyme loading of 40 mg protein/g cellulose. Protein concentration was determined using Pierce’s BCA protein assay kit (Thermo Fisher Scientific, Rockford, IL), with bovine serum albumin as the protein standard. Enzymatic hydrolysis runs at five different insoluble solids loadings (ranging from 15 to 25 % insoluble solids) were performed.

### Enzymatic Hydrolysis of Soluble Xylo-oligomers in Pretreated Corn Stover Hydrolyzate

All enzymatic saccharifications of primary pretreatment hydrolyzate liquor were performed at 50 °C in 1.5-mL Eppendorf tubes in 100-mM citrate buffer at pH 4.8 with sodium azide added at 0.001 % to prevent microbial growth. Separate saccharifications of the hydrolyzate liquid fractions were performed at dilution factors of 1.3×, 10×, and 50× to test the impact of product inhibition caused by high background sugar concentrations. For the 1.3× dilution series, an additional 100 μL of 0.1 M NaOH was added to the total volume. All hydrolyzate saccharifications were run for 24 h.

Enzymes used to perform hydrolyzate saccharifications were the commercial xylanase preparations Multifect Xylanase from Genencor Inc. (Rochester, NY) and a selection of other purified enzymes. Purified beta-xylosidase (XlnD) from *Aspergillus niger* was used to ensure conversion of all simple xylo-oligomers to monomer xylose [18]. Additional accessory enzymes that were used in the “loaded enzyme mix” preparation included *A. niger* arabinofuranosidase (AF), *Trichoderma reesei* acetyl xylan esterase (Axe), and *Aspergillus clavatus* alpha-glucuronidase loaded at rates of 5, 5, and 1 mg/g XO, respectively [17]. All enzymes were purified to >95 % homogeneity by protein chromatography.

### Consecutive Enzymatic Saccharification and Fermentation of Pretreated Corn Stover Whole Slurry

The pretreated whole slurry was neutralized to a pH of 4.8 with ammonium hydroxide. Enzymatic hydrolysis was conducted at 15, 17.5, and 20 % total solids loadings using Novozymes Cellic CTec at an enzyme loading of 40 mg protein/g cellulose. Enzymatic hydrolysis was performed with 1,000 g pretreated slurry in 2-L capped bottles in a shaking incubator at 150 rpm for 120 h at 48 °C and a pH of 4.8.

After enzymatic saccharification was complete, fermentation was conducted in Sartorius Stedim (formerly B. Braun Biotech, Aubagne, France), BioStat-Q-plus fermenters at a 400-mL working volume using *Zymomonas mobilis* strain 8b. A rich medium consisting of 10 g/L yeast extract, 2 g/L KH_2_PO_4_, and enzymatically hydrolyzed whole slurry as described above was added to the fermenters, which were then inoculated at an optical density of ∼1.0 absorbance units (at 600 nm) using a (10 % *v*/*v*) inoculum. The pH was automatically controlled at 5.8 using 3 M potassium hydroxide (KOH); agitation was controlled at 300 rpm at a temperature of 33 °C for 72 h. Sugar conversion and ethanol yield calculations were based on initial and final glucose, xylose, fructose, and ethanol concentrations. The post-fermentation samples were stored frozen until used in subsequent experiments to determine if any available residual xylo-oligomers could be converted under various enzymatic xylo-oligomer conversion strategies.

### Enzymatic Hydrolysis of Soluble Xylo-oligomers in Post-fermentation Broth

To determine whether conversion of residual xylo-oligomers was enhanced after background monomeric sugars were consumed and converted to ethanol during fermentation, samples of post-fermentation broth were generated as described above.

Soluble xylo-oligomers remaining from the whole slurry enzymatic saccharification and fermentation experiments were subjected to a secondary enzymatic hydrolysis using targeted xylanase and purified “accessory” enzyme activities. Enzyme sources and procedural details were detailed above under “Enzymatic Conversion of Xylo-oligomers to Monomeric Xylose.” Three post-fermentation slurries, originally prepared with 15, 17.5, and 20 % (*w*/*w*) total solids, were used in these experiments. Yields of additional monomeric xylose were determined using the available xylo-oligomer content as the yield basis.

### Analytical

The composition of raw, acid-impregnated, and hydrolyzed corn stover was measured using a standard set of procedures (NREL) for determining carbohydrate, acid insoluble lignin, acid soluble lignin, ash, and acetate content [19, 21]. Concentrations of monomeric xylose, oligomeric xylose, and furfural (xylose degradation product) of the pretreated corn stover hydrolyzate liquor were determined, along with total solids content and insoluble solids content [19]. Acid concentration of pre-impregnated corn stover was determined by blending 10 g of impregnated stover into 230 g deionized water for 3 min followed by soaking for 45 min in a Waring commercial laboratory blender. Fifty grams of filtered liquid was titrated using a Mettler Toledo Model T70 Automated Titrator with 1 N NaOH (Fisher Scientific certified 1.005–0.995 N). A Mettler Toledo XS4002S laboratory balance was used to weigh impregnated corn stover and deionized water, and a Mettler Toledo AE200 laboratory balance was used to weigh titration samples.

Monomeric and oligomeric xylose concentrations in hydrolyzate liquor enzymatic hydrolysis samples and the post-fermentation enzymatic hydrolysis samples were determined using NREL’s standard laboratory analytical procedures for soluble carbohydrate measurements [19]. These concentrations served as the basis for determining yields and monomeric xylose produced from available xylo-oligomers.

## Results and Discussion

After pretreatment and subsequent secondary mild thermochemical oligomer hydrolysis, 7 % of the original xylan remains insoluble in the pretreated solids. While further attempts to thermochemically solubilize more xylan produce increasing levels of degradation products, enzymatic conversion offers the potential to hydrolyze insoluble xylan with no further degradation.

### Enzymatic Hydrolysis of Washed Pretreated Corn Stover Solids

Results from the enzymatic hydrolysis of pretreated washed solids using Novozymes Cellic CTec2 are shown in Table 3.

**Table 3:** Yield of monomeric sugars achieved during the enzymatic hydrolysis of washed dilute acid-pretreated corn stover using Novozymes Cellic CTec2

Slurry solids loading (%)	Cellulose to glucose yield (%)	Xylan to xylose enzymatic hydrolysis yield (%)
15.0	96.0	85.9
20.0	94.5	81.4
25.0	88.5	82.8

For all of the solids loadings investigated, over 80 % of the insoluble xylan was converted to xylose, with an overall average of ∼83 % xylose yield using a commercially available cellulase enzyme cocktail. Thus, significant potential exists to improve overall xylose yields through the enzymatic hydrolysis of xylan remaining after pretreatment. Further improvements can be expected with the next generation of cellulase cocktails and the inclusion of hemicellulose-targeted mixtures.

Initial solids loading had the expected impact on solids loading, as 96 % of cellulose was converted to glucose at 15 % solids, and only 88.5 % was converted at 20 % solids.

### Enzymatic Hydrolysis of Soluble Xylo-oligomers in Pretreated Corn Stover Hydrolyzate

Preliminary studies investigating high-solids enzymatic hydrolysis have found significant inhibitory effects on enzyme activity caused by high concentrations of background sugars present in the pretreated hydrolyzate [24]. Consequently, experiments were performed to determine the impact of the high product concentrations on the conversion of xylo-oligomers using a commercial enzyme cocktail. Results are depicted in Table 4.

**Table 4:** Enzymatic conversion of xylo-oligomers to monomeric xylose using Multifect Xylanase at a loading of 20 mg protein per gram of xylo-oligomer

Dilution factor	Initial xylose concentration (g/L)	Corresponding equivalent solids (%)	Conversion (%)
1.3	68.46	23	2.3
10.0	8.90	3	44.3
50.0	1.78	1	43.1

Background xylose concentrations of over 8.90 g/L have inhibitory effects on enzyme activity

Dilution of the background xylose concentration to less than 9 g/L improved enzyme activity by more than ten times, achieving conversion of nearly 45 % of xylo-oligomers to monomeric xylose. These experiments are done on the liquor fraction of pretreated corn stover to ensure that newly formed xylose generated from xylo-oligomers is not confounded with xylose from the insoluble solids fraction of pretreated slurry. However, these experimental conditions can be interpreted in terms of equivalent percent solids. The three dilution levels utilized (1.3×, 10×, and 50×) have equivalent soluble carbohydrate concentrations to a 30 % solids whole slurry that is diluted to solids loadings of 23, 3, and 1 %.

As past work has shown, still higher yields can be achieved through supplementing basic xylanase cocktails with accessory enzymes targeting side chains commonly found on xylan [17, 18]. Common substitutions include arabinosyl and acetyl groups, and some of the most recalcitrant oligomers are 4-*O*-methylglucuronic acids. Acetyl xylan esterase, arabinofuranosidase, and alpha-glucuronidase were added to Genencor Multifect Xylanase to target these units and presumably allow the xylanase enzymes better access. Beta xylosidase was also supplemented to ensure complete conversion of simple xylo-oligomers to xylose.

**Fig. 3:**
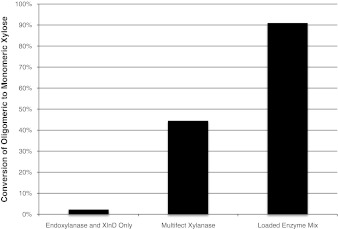
Supplementation of Multifect Xylanase with debranching enzymes targeting known side-chains significantly improve enzymatic conversion of process-generated xylo-oligomers. Little to no conversion was observed with endoxylanase and beta-xylosidase only

Adding these accessory enzymes to Multifect Xylanase in liquor containing sugar concentrations equivalent to 3 % solids converted more than 90 % of xylo-oligomers to monomeric xylose, as depicted in Fig. 3.

The high conversion of xylo-oligomers to monomeric xylose when using the “loaded” enzyme mix demonstrates the positive impact of removing inhibitory side chains by accessory enzymes with only a modest increase in total enzyme loading. The loaded enzyme mix, as implemented in these experiments, comprised only 6 % of the cellulase loading at a level of 20 mg/g. However, replicating these levels of dilution in a whole slurry context would require dilution to 3 % solids, which would not be feasible in either the pilot- or commercial-scale processes. At such scale, minimal dilution will be permissible, but similarly low sugar concentrations will be present in the last 24 h of batch fermentation. To mimic the loss of xylose during fermentation, a 24-h time-course dilution experiment was designed wherein the initial sample was diluted to 23 % solids and ultimately was diluted to 3 % solids over 24 h.

**Fig. 4:**
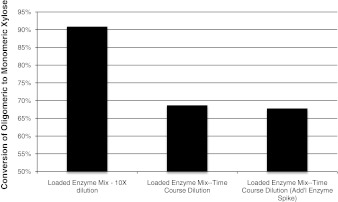
Dilution by 11.7X over a period of 24 h achieved nearly 70 % conversion of xylo-oligomers to monomeric xylose. Addition of additional enzymes at the final dilution stage found no significant conversion improvements over samples where enzymes were added only initially

In the time-course dilution experiment, nearly 70 % conversion of oligomeric to monomeric xylose was achieved using the loaded enzyme mix, as shown in Fig. 4. In another experiment, an additional dose of enzymes was added when the terminal dilution was achieved after 6 h. These were added in the event that the enzymes added initially were permanently deactivated by the high starting sugar concentrations. However, the secondary addition did not appear to have any significant effect.

While the high conversion of xylo-oligomers is encouraging, several issues were not modeled in this experiment. For this hydrolysis to occur when sugar concentrations drop in late-stage fermentation, as simulated in this experiment, enzymes would have to maintain activity at the pH and temperature optimums for the fermentation organism. Additionally, fermentation products such as ethanol were not included in this experiment and could possibly act as enzyme inhibitors.

### Enzymatic Hydrolysis of Soluble Xylo-oligomers in Post-fermentation Broth

To evaluate the inhibitory effects of fermentation products, the loaded enzyme mixture was used to convert the xylo-oligomers remaining after whole slurry fermentation. Several fermentation broths were selected, with varying pre-fermentation solids loadings and, thus, different initial sugar concentrations (Table 5).

**Table 5:** Yield of monomeric sugars from xylo-oligomers contained in fermentation broth upon subsequent residual xylo-oligomer enzymatic hydrolysis using xylanase and accessory enzyme cocktails

Initial slurry solids loading (*w*/*w*)	Treatment	Pre-dilution xylose concentration (g/L)	Xylose yield from xylo-oligomers (%)
15.0	1:2 dilution with enzyme mix	4.2	9.6
	1:20 dilution with enzyme mix	4.9	29.9
17.5	1:2 dilution with enzyme mix	6.6	0.0
	1:20 dilution with enzyme mix	8.3	38.1
20.0	1:2 dilution with enzyme mix	18.3	0.0
	1:20 dilution with enzyme mix	22.2	59.1

Thirty percent (30 %) to 60 % of xylo-oligomers were converted enzymatically in fermentation broth diluted 1:20. However, in samples diluted 1:2, no significant conversion was observed. The depressed enzyme activity at low dilution levels is most probably caused by significant ethanol concentrations of approximately 30 g/L. The positive correlation between solids loading and yield at the 1:20 dilution level is likely due to increasing quantity of solids present and the hydrolysis of insoluble xylan remaining in those solids.

Significant potential exists to enzymatically convert process generated xylo-oligomers to monomeric xylose. However, techniques must be developed to overcome the inhibitory effects of ethanol and other fermentation products on xylanases and other accessory enzymes. The required enzyme addition and conversion should not add significant cost to the process, as utilization of the loaded enzyme mix described in this work would only comprise 6 % of cellulase loaded at 20 mg/g cellulose.

The enzymatic conversion of insoluble xylan and xylo-oligomers to monomeric xylose has been demonstrated to have the potential to improve process economics through the improvement of xylan-to-xylose yield. While these results are promising, conversion using pretreated whole slurry rather than washed solids needs to be evaluated. The exhaustive washing stage required is energy and water intensive, and thus costly on the process scale. If similar conversion yields can be achieved using whole slurry, these improvements would be greatly beneficial to process economics.

## Conclusions

Up to 70 % of xylo-oligomers and 80 % of insoluble xylan in dilute acid-pretreated stover can be converted to monomeric xylose using a viable enzyme cocktail. The overall improvement in xylan-to-xylose yield can be expected to greatly improve process economics, as only a modest increase in enzyme dosage is required, only 6 % of the cellulase enzyme loading. Demonstration of high yields of xylose from xylo-oligomers and xylan in an enzymatic conversion step may lead to lower severity pretreatments, which reduces formation of sugar degradation products, and other fermentation inhibitors. The caveat is that high concentrations of fermentation and hydrolysis products may inhibit enzyme activity.
